# Urgent Admission and Inequities in Acute Hospital Stay in Canada

**DOI:** 10.3390/ijerph23040432

**Published:** 2026-03-30

**Authors:** Kisalaya Basu

**Affiliations:** Health System Policy and Analytic Division, Health Policy Branch, Health Canada, Ottawa, ON K1A 0K9, Canada; bkisalaya@gmail.com or kisalaya.basu@hc-sc.gc.ca

**Keywords:** urgent admission, rural–urban residence, socioeconomic status, length of stay, hospital resource utilization, health equity

## Abstract

**Highlights:**

**Public health relevance—How does this work relate to a public health issue?**

- This study examines how admission urgency modifies socioeconomic and geographic inequities in hospital length of stay within Canada’s universal healthcare system.
- It highlights how barriers to timely access and care continuity influence downstream hospital utilization during acute illness.

**Public health significance—Why is this work of significance to public health?**

- The findings show that universal coverage for hospital care does not eliminate inequities, but alters how and where they manifest along the care pathway.
- By linking access limitations to prolonged hospitalization under urgent care, the study identifies a key mechanism through which upstream inequities burden acute care systems.

**Public health implications—What are the key implications or messages for practitioners, policy makers and/or researchers in public health?**

- Efforts to reduce hospital length of stay should prioritize improving timely access, prevention, and care coordination before conditions become urgent.
- Public health policies focused solely on inpatient efficiency are unlikely to reduce inequities without parallel investments in upstream and community-based care.

**Abstract:**

Background: The Canada Health Act (CHA), enacted in 1984, guarantees universal access to medically necessary care, yet inequities in hospital use persist. Acute length of stay (ALOS) is a key indicator of hospital efficiency, patient recovery, and healthcare system performance, with prolonged stays linked to higher costs, avoidable infections, and strain on acute care capacity. Understanding patterns in ALOS is critical not only for hospital management but also for public health, as extended stays can limit timely access to care and exacerbate population-level health inequities. Objective: This study examines social, geographic, and clinical gradients in ALOS and investigates whether the effects of admission urgency vary by sex, neighbourhood income, and rural–urban residence within a universal healthcare system. Methods: Using 2024–2025 hospital discharge data from the Canadian Institute for Health Information, this study examined ALOS as a function of comorbidity, sex, socioeconomic status, rural–urban residence (geography), and admission type (urgent versus elective). Interaction effects between admission urgency and key social and geographic variables were evaluated to assess subgroup differences in ALOS. Results: Disparities in ALOS were evident. Older age, male sex, urgent admission, and greater comorbidity were associated with longer stays, whereas higher neighbourhood income and urban residence were linked to shorter stays. Interaction analyses revealed substantial heterogeneity: compared with elective rural admissions, urgent urban admissions had 30.4% longer ALOS. Urgent admissions also amplified socioeconomic and sex-based differences, with male patients experiencing 27.9% longer stays than females. Conclusions: From a public health perspective, these findings highlight how system capacity constraints and social inequities jointly shape hospital use. Reducing avoidable variation in ALOS will require policies that strengthen acute care surge capacity, improve coordination for urgent admissions, and address upstream socioeconomic and geographic barriers to care, thereby promoting more equitable and efficient hospital services.

## 1. Introduction

The Canada Health Act (CHA), enacted in 1984, is the foundation of Canada’s publicly funded healthcare system and a key instrument for promoting population health equity. It guarantees reasonable access to medically necessary hospital and physician services without direct charges, guided by the principles of public administration, comprehensiveness, universality, portability, and accessibility [1]. By design, the CHA aims to ensure that care is delivered based on need rather than ability to pay, regardless of socioeconomic status or geography. However, persistent disparities in hospital use indicate that non-financial barriers continue to shape access, utilization, and outcomes.

Acute length of stay (ALOS) is a widely used indicator of hospital resource use and health system performance [2]. In this study, ALOS refers specifically to the number of days during which a patient requires active acute care treatment. This differs from total length of stay, which may also include additional days spent in hospital after acute treatment is completed while the patient awaits discharge to an appropriate destination, such as long-term care or rehabilitation. From a public health perspective, ALOS reflects not only clinical recovery but also system efficiency, care coordination, and equity in service delivery. Prolonged hospital stays are associated with higher costs, increased risk of hospital-acquired infections, delayed patient flow, and lower patient satisfaction [3]. Established determinants of ALOS include clinical factors such as comorbidity burden and illness severity, as well as demographic characteristics including age and sex. Increasingly, evidence suggests that social and geographic conditions also play a critical role in shaping hospital stays.

Rural–urban disparities are a persistent feature of Canada’s health system. Rural residents experience higher rates of hospitalization and emergency department use than urban populations, reflecting differences in access to primary care, specialist services, and diagnostic capacity. For example, rural patients with inflammatory bowel disease have higher hospitalization rates despite comparable outpatient outcomes, partly due to limited specialist access [4]. Similarly, rural head and neck cancer patients in Alberta, Canada, had greater postoperative resource use than urban counterparts [5]. Similar patterns have been observed in other conditions, indicating that structural barriers and care coordination challenges can prolong hospital use in rural settings.

Socioeconomic gradients in hospital outcomes are also well documented. Lower-income individuals are more likely to delay care, present with more advanced illness, and face constraints in post-discharge support, contributing to longer hospital stays. Evidence from Quebec shows that trauma patients from deprived neighborhoods had significantly longer length of stay than those from wealthier areas, even after adjusting for injury severity [6].

Evidence on sex differences in hospital length of stay remains mixed and context-dependent, varying across clinical settings and care contexts. Male patients often present with higher acuity, greater comorbidity burden, and older age, contributing to longer hospital stays [7]. In critical care, women generally have shorter ICU stays than men [8], while Canadian stroke rehabilitation data show only marginal sex differences in length of stay, with females experiencing slightly longer stays due to age and post-discharge needs [9]. A US study reported that women experienced longer hospital stays and higher risk of complications, including hypovolemic shock and need for transfusion [10]. These findings indicate that sex alone does not fully explain length of stay differences, and that factors such as age, social conditions, and admission context are important. However, few studies have examined how sex interacts with admission urgency, highlighting an important gap in the literature [7,8,9,10].

Admission urgency adds another important dimension. Urgent admissions differ from elective admissions in clinical severity, resource intensity, and discharge planning, and may reflect upstream gaps in prevention, primary care access, and chronic disease management. Despite its relevance for population health and system performance, limited national evidence exists on how social and geographic factors modify the relationship between admission urgency and ALOS.

This study addresses this gap by examining how admission urgency interacts with socioeconomic status, sex, and rural–urban residence to shape ALOS in Canada. By focusing on the intersection of clinical urgency with social and geographic conditions, the analysis highlights how inequities in hospital use can persist even within a universal healthcare system. Understanding these patterns is critical from a public health perspective, as avoidable variation in hospital length of stay reflects upstream gaps in access, care coordination, and system capacity. The findings aim to inform equity-oriented policies that improve timely access to care, strengthen coordination across the care continuum, and enhance the efficiency and fairness of hospital services in Canada.

The paper proceeds with a data description, followed by methodology, findings, discussion, limitations, and conclusions.

## 2. Data Sources

This study used data from the Discharge Abstract Database (DAD) for the 2024–2025 fiscal year, obtained from the Canadian Institute for Health Information (CIHI). The DAD is a national administrative database capturing all acute care hospital discharges in Canada, excluding Quebec, and is widely used in health services research to assess hospital utilization, system performance, and health inequities.

The database contains standardized information on admission characteristics, including admission urgency, discharge destination, and alternate level of care status; clinical factors such as primary diagnosis and comorbidities; demographic attributes including age and sex; geographic indicators at the provincial and rural–urban levels; and socioeconomic status measured using neighbourhood income quintiles. Its population-based coverage, national scope, and large sample size provide high statistical power and enable precise estimation of associations [11].

The DAD is particularly well suited to this analysis because it allows admissions to be classified by urgency and examined in relation to socioeconomic and geographic context, enabling robust assessment of disparities in hospital utilization across neighbourhood income groups and rural–urban settings.

## 3. Methodology

### 3.1. Exclusionary Criteria

First, Quebec was excluded due to data limitations, and the three territories were omitted because of their small sample sizes within the cohort. Patients discharged to another acute or subacute facility were also excluded because the database does not track the duration of their subsequent stay in those facilities. Additional exclusions included patients who died in hospital or while on leave, did not return from leave, left against medical advice, received medical assistance in dying, or died by suicide outside a facility.

Second, primary diagnoses were classified using the *International Statistical Classification of Diseases and Related Health Problems, 10th Revision* (ICD-10; WHO, 2016) [12]. Patients with diagnoses representing very small sample sizes were excluded, including diseases of the eye and adnexa (0.17%), ear and mastoid process (0.23%), congenital malformations and chromosomal abnormalities (0.41%), and perinatal conditions (0.81%). Cases related to pregnancy, childbirth, and the puerperium (13.56%) were also excluded, as they apply only to female patients.

### 3.2. Econometric Model

Acute length of stay, measured in days, is a count outcome that exhibits overdispersion, with variance exceeding the mean. Accordingly, I used a negative binomial regression model, which provides more reliable estimates than a Poisson model under conditions of overdispersion. This approach is well suited for modeling count outcomes with non-normal and heteroskedastic distributions and allows for robust estimation of associations between patient characteristics and ALOS.

The expected ALOS for patient i was specified as:

(1) $$ln\left(\mu_{i}\right)=\beta_{0}+\beta_{1}A_{i}+\beta_{2}U_{i}+\sum_{k=2}^{5}\beta_{k+1}Q_{ki}+\beta_{7}M_{i}+\;\beta_{8}\left(A_{i}\times U_{i}\right)+\sum_{k=2}^{5}\beta_{8+k-1}\left(A_{i}\times \;Q_{ki}\right)+\beta_{13}\left(A_{i}\times M_{i}\right)+\;Z_{i}^{\prime }\;y_{i}\sim NegBin\left(\mu_{i},\;\alpha \right)$$

Here, $\mu_{i}$ denotes expected ALOS, while $y_{i}$ is the observed ALOS for patient i. Admission type is captures by $A_{i}$ (1 = urgent, 0 = elective); area by $U_{i}$ (1 = urban, 0 = rural); neighbourhood income quintile indicators $Q_{ki\;}$ with the lowest quintile (k=1) as the reference; and sex $M_{i\;}$ (1 = male, 0 = female). Control variables $\left(Z_{i}\right)$ include age, number of comorbid conditions, province dummies (Provinces are Newfoundland, Prince Edward Island, Nova Scotia, New Brunswick, Ontario (reference), Manitoba, Saskatchewan, Alberta, and British Columbia; Quebec was not included as the data was not available), and primary diagnosis dummies. Using a count of comorbidities is acceptable and appears in the literature, especially when the goal is to capture overall morbidity burden, even though indices like the Charlson Comorbidity Index or Elixhauser Comorbidity Measure may provide more refined risk adjustment. 

The DAD includes 25 ICD-10 diagnosis fields: the first records the primary diagnosis, while the remaining 24 capture comorbid conditions. ICD-10 codes beginning with V, W, X, and Y—used to describe external causes or contexts of injury (e.g., mechanism, intent, or location)—do not represent clinical diagnoses and are not intended as primary diagnoses. To avoid double-counting and ensure an accurate comorbidity count, these codes were excluded. The 15 broad diagnostic categories used in this study include: a. Infectious and parasitic diseases; b. Neoplasms; c. Diseases of the blood; d. Endocrine and related diseases; e. Mental and behavioral disorders (reference); f. Diseases of the nervous system; g. Diseases of the circulatory system; h. Diseases of the respiratory system; i. Diseases of the digestive system; j. Diseases of the skin and subcutaneous tissue; k. Diseases of the musculoskeletal system and connective tissue; l. Diseases of the genitourinary system; m. Symptoms, signs, and abnormal clinical and laboratory findings; n. Injury and poisoning; and o. Factor influencing health status. The parameter α represents the overdispersion of the negative binomial distribution.

The choice of explanatory variables is informed by the Andersen’s Behavioral Model of Health Services Use, which classifies determinants of healthcare utilization into predisposing, enabling, and need factors. Predisposing characteristics include age and sex, which capture demographic differences in health status and healthcare use. Enabling factors include neighbourhood income quintile, urban-rural residence, and provincial indicators, reflecting socioeconomic and geographic variation in access to healthcare resources. Need factors are represented by admission type, the number of comorbid conditions, and primary diagnosis categories, which capture the severity and nature of illness that generate demand for hospital services. In the context of Canada’s publicly funded healthcare system, governed by the Canada Health Act, hospital utilization is expected to be driven primarily by clinical need, while the inclusion of enabling factors allows for the assessment of potential socioeconomic and geographic differences in patterns of hospital care.

Model results are reported as incidence rate ratios (IRRs), which were converted to percentage changes using the formula:

$$\%\;change=\left(IRR-1\right)\times 100$$

IRRs represent the proportional change in expected ALOS associated with a one-unit change in a predictor, holding other variables constant. For example, an IRR of 1.20 indicates an expected stay that is 20% longer than the reference group, whereas an IRR of 0.80 indicates an expected stay that is 20% shorter. For interaction terms, combined IRRs capture the joint effect of the main and interaction coefficients relative to the reference group. Specifically, for the admission × rural–urban interaction, the reference group is elective admissions in rural areas. For the admission × income quintile interactions, the reference group is elective admissions in quintile 1. For the admission × sex interaction, the reference group is elective admissions among females.

Statistical significance was evaluated at the 1% level, and all analyses were conducted in SAS 9.4 (SAS Institute Inc., Cary, NC, USA).

## 4. Empirical Findings

### 4.1. Summary Statistics

Table 1 presents descriptive statistics for the continuous variables included in the regression models. On average, patients were 60.7 years old, had 3.21 comorbid conditions, and stayed 7.1 days in hospital. The standard deviation of comorbidities was nearly equal to the mean, indicating substantial variability in patients’ morbidity burden. Similarly, the standard deviation of ALOS exceeds the mean, suggesting a right-skewed distribution in which most patients experience relatively short stays while a smaller proportion remain hospitalized for much longer periods.

Table 2 presents the distribution of categorical variables used in the regression analyses. Among inpatients, 50.2% were female and 49.8% were male. Rural residents accounted for 19.4% of patients, while 80.6% lived in urban areas. The distribution across income quintiles (1–5) was 27.0%, 21.9%, 18.6%, 16.8%, and 15.7%, respectively. The four most common primary diagnoses were diseases of the circulatory system (14.2%), diseases of the digestive system (12.6%), diseases of the respiratory system (10.7%), and injury and poisoning (10.4%).

Additional descriptive statistics stratified by admission type (urgent vs. elective) are provided in Appendix A Table A1 and Table A2 to illustrate subgroup differences.

### 4.2. Results from the Negative Binomial Regression Model

Table 3 presents the results of the negative binomial regression model, which includes interaction terms between admission type and income quintile, rural–urban residence, and sex to estimate ALOS. All coefficients are statistically significant at the 1% level. The last two columns report the IRRs and the combined IRRs for the interaction terms, where the latter reflect the multiplicative effect obtained by combining the main effect with its corresponding interaction.

Multicollinearity among predictors was assessed using variance inflation factors (VIFs). All VIFs were below 10, with most under 5 and two between 7 and 9, indicating moderate correlation for those two variables. While such values may slightly inflate standard errors, they are generally not considered problematic for model estimation or inference. Given the theoretical importance of the predictors and the stability of coefficient estimates, all variables were retained in the analysis.

#### 4.2.1. Main Effects

Controlling for other factors, older age, male sex, urgent admission, and a higher number of comorbidities were all associated with longer ALOS, whereas urban residence was linked to slightly shorter stays. Specifically, each additional year of age increased the expected stay by 0.4%, and each additional comorbid condition by 18.0%. Urgent admissions were associated with a 32.0% longer stay compared with elective admissions. Male patients stayed 9.9% more than females, and urban residents 6.1% less than rural residents. Income also played a role: compared with the lowest quintile, patients in quintiles 2 through 5 had progressively lower stays by 4.8%, 6.5%, 9.3%, and 12.3%, respectively (all *p* < 0.01). Considerable variation was observed across provinces.

#### 4.2.2. Interaction Effects

After controlling for all other variables, several significant interaction effects emerged. Using elective admissions in rural areas as the reference group, urgent admissions in urban areas were associated with an expected ALOS that was 30.4% longer (IRR = 1.304, *p* < 0.01). Notably, this result contrasts with the main effect of urban residence, which was associated with shorter stays, indicating that the baseline urban advantage in ALOS is substantially attenuated and reversed under urgent, high-acuity admissions.

Sex also modified the relationship between admission type and ALOS. Male patients admitted urgently experienced hospital stays that were 27.9% longer than the reference group (IRR = 1.279, *p* < 0.01). Income further moderated the effect of admission urgency: relative to elective admissions in income quintile 1, urgent admissions were associated with longer expected stays by 29.7% in quintile 2 (IRR = 1.296, *p* < 0.01), 28.5% in quintile 3 (IRR = 1.285, *p* < 0.01), 27.9% in quintile 4 (IRR = 1.279, *p* < 0.01), and 27.3% in quintile 5 (IRR = 1.273, *p* < 0.01).

Taken together, these interaction effects indicate that while urban residence and higher income are associated with shorter ALOS in the main effects, the advantages linked to urban residence are not preserved under urgent admission, whereas socioeconomic and sex-based differences persist in shaping the magnitude of ALOS during acute hospitalizations.

#### 4.2.3. Predicted Adjusted ALOS Values for Key Subgroups

Adjusted ALOS estimates revealed important differences across patient subgroups (Table 4). Male patients had longer hospital stays than females, and rural residents experienced longer stays than urban residents. Higher-income patients had progressively shorter stays compared with those in lower-income quintiles. As expected, urgent admissions were associated with substantially longer ALOS than elective admissions, and additional comorbid conditions further increased expected stays.

When interaction effects were considered (Table 5), these patterns were modified by admission urgency. The difference in ALOS between males and females was reduced for urgent admissions. Similarly, the shorter stays observed among urban residents and higher-income patients under elective care were attenuated in urgent settings. Although urgent admissions increased ALOS across all subgroups, the magnitude of this increase varied, indicating that clinical urgency can partially offset socioeconomic and geographic differences in hospital utilization.

Together, these findings highlight the joint influence of predisposing characteristics, enabling factors, and clinical need, consistent with Andersen’s Behavioral Model of Health Services Use, and underscore how both patient- and system-level factors shape hospital utilization in Canada’s publicly funded healthcare system.

Table 5 presents adjusted ALOS values for key interaction subgroups, highlighting how admission urgency modifies the effect of sex, area, and income on hospital stays. Urgent admissions were associated with substantially longer stays across all subgroups, but the magnitude of this effect varied. Male patients generally had longer stays than females, though the difference was slightly reduced for urgent admissions. Urban residents experienced shorter stays than rural residents for elective admissions; however, this pattern was reversed for urgent admissions, with urban patients experiencing longer stays. Similarly, higher-income patients had progressively shorter stays compared with lower-income patients for elective admissions, but the income gradient narrowed for urgent admissions. These findings indicate that clinical urgency can attenuate disparities related to sex, socioeconomic status, and geographic residence, while still reflecting persistent differences in baseline hospital utilization across subgroups.

#### 4.2.4. Predicted Acute Length of Stay by Key Patient Subgroups and Admission Urgency

**Figure 1:**
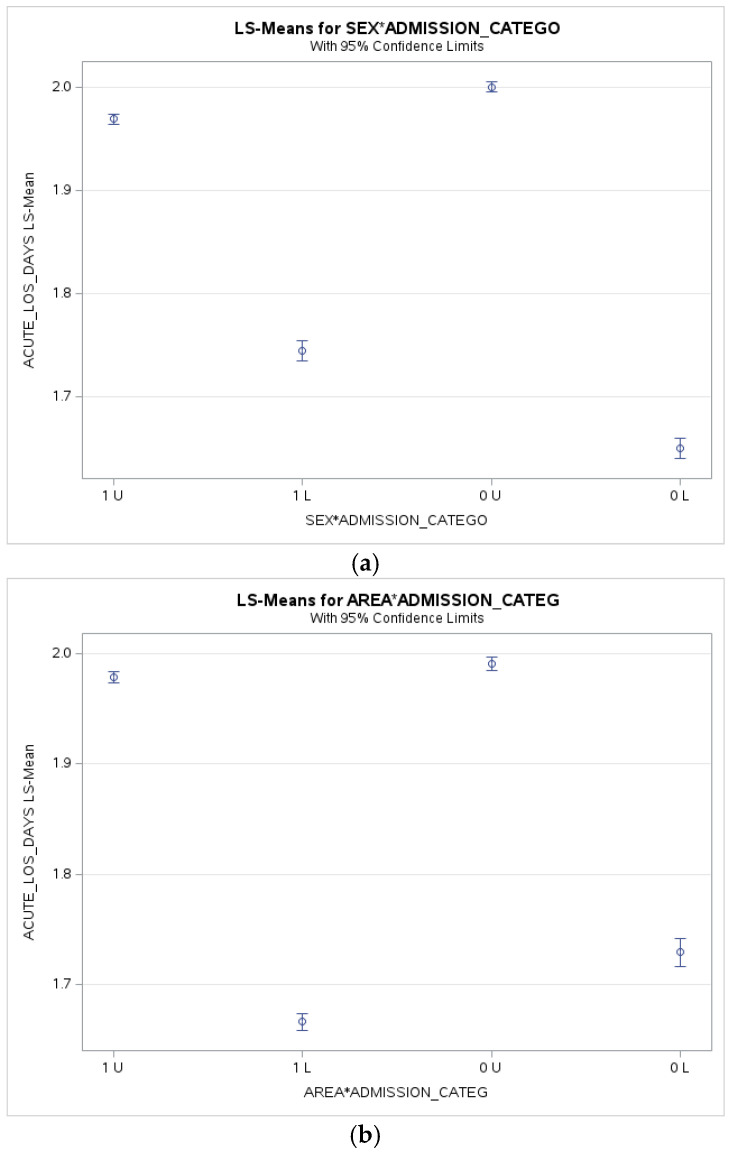

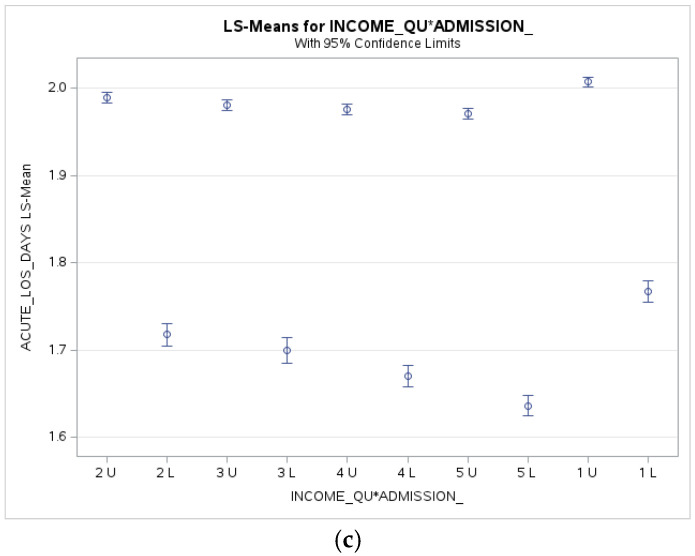
(**a**) Adjusted Acute Length of Stay by Sex and Admission Category; (**b**) Adjusted Acute Length of Stay by area and Admission Category; (**c**) Adjusted Acute Length of Stay by Neighbourhood Income Quintile and Admission Category; *: implies interaction.

Figure 1a–c present predicted ALOS across key patient subgroups, illustrating how sex, area of residence, and neighbourhood income interact with admission urgency. These marginal effect plots highlight subgroup-specific differences in hospital stays while controlling for age, comorbidities, province, and primary diagnosis.

## 5. Discussion

This study demonstrates that ALOS in Canadian hospitals is shaped by the combined influence of clinical, socioeconomic, and geographic factors, with important implications for public health and healthcare system performance. Among clinical determinants, older age, higher comorbidity burden, and urgent admission were the strongest predictors of prolonged stays. Each additional comorbidity increased ALOS by 18%, underscoring how patient complexity places sustained pressure on acute care capacity and highlights the importance of effective chronic disease management as a public health priority.

Non-clinical factors also played a meaningful role. Men experienced 9.9% longer stays than women, consistent with evidence of sex-based differences in health-seeking behaviour, disease severity at presentation, and recovery trajectories. Lower engagement with preventive and primary care among men may contribute to delayed presentation and more advanced illness at admission, complicating inpatient management. Urban residence, in contrast, was associated with shorter stays in the main effects, likely reflecting better access to diagnostic services, specialist care, and post-discharge supports. Similar urban advantages have been observed among heart failure patients, who show lower hospitalization and emergency department use [13].

Interaction analyses show that admission urgency fundamentally alters how patient geography and socioeconomic status shape ALOS. While urban residence and higher income were associated with shorter stays under elective care, these advantages diminished once admissions became urgent. Among patients residing in urban areas, urgent admissions were associated with substantially longer stays, and income-related differences narrowed under urgent care. For rural residents, high-acuity episodes often involve referral or transfer to urban hospitals for specialized care, whereas urban residents are less likely to experience comparable transfers. As a result, urgent admissions tend to concentrate clinically complex patients from both rural and urban backgrounds within the same high-volume care settings. Under these conditions, advantages linked to urban residence and higher socioeconomic status, such as better access to outpatient services and coordinated elective care pathways, offer limited protection. Delays in presentation, increased illness severity at admission, and challenges in coordinating care transitions across settings may prolong recovery, offsetting efficiencies observed under routine care.

Findings also suggest that both predisposing and need factors, as described by Andersen’s Behavioral Model, continue to influence hospital length of stay in Canada’s publicly funded system. Male patients and those living in rural areas tended to have longer stays, while urban residents experienced shorter stays, indicating that demographic and geographic characteristics shape utilization even when financial barriers are minimized. The effect of urgent admission was modified by sex, with male patients experiencing proportionally longer stays when admitted urgently. This may reflect that males are more likely to seek care promptly in response to acute illness, resulting in more severe presentations at admission. Overall, these results highlight that, even in a universal system, individual characteristics and admission context remain important drivers of hospital utilization, with implications for resource planning and equitable care delivery.

Findings of this study indicate that geography is associated with differences in hospital utilization. Urban residents generally experienced shorter stays than rural patients, likely reflecting stronger access to specialist care, post-discharge supports, and community-based services that facilitate timely discharge. However, this advantage appears to weaken in high-acuity situations. When admissions occur on an urgent basis, urban patients experience comparatively longer stays than the reference group, suggesting that emergency demand, hospital crowding, and coordination challenges may offset the structural benefits of urban health systems. These patterns align with the Andersen’s Behavioral Model of Health Services Use, which highlights how enabling factors such as geographic access and need factors such as illness severity jointly shape healthcare utilization, even within Canada’s publicly funded system under the Canada Health Act.

From a public health perspective, these findings underscore the limits of downstream hospital care in compensating for upstream inequities in access, prevention, and continuity of care. Policies focused solely on inpatient efficiency are therefore unlikely to reduce disparities that originate earlier in the care pathway.

At the same time, rural and peri-urban residents continue to face persistent structural barriers, including limited access to family physicians and specialists and greater reliance on emergency departments, even when hospitalization rates are comparable [14]. Socioeconomic gradients further reinforced inequities, with patients in higher income quintiles experiencing up to a 12.3% reduction in ALOS compared with those in the lowest quintile. These findings align with broader evidence linking lower socioeconomic status to longer hospital stays and higher hospitalization risk [6,15,16].

Consistent with national evidence on rural health inequities, rural communities account for approximately 18% of Canada’s population yet are served by a disproportionately small share of physicians. Persistent challenges in recruiting and retaining healthcare providers have been associated with higher hospitalization rates and greater costs from potentially avoidable admissions for ambulatory care sensitive conditions, particularly among low-income and lower-education populations [17]. Similar patterns have been observed among rural patients with inflammatory bowel disease, who experience higher hospitalization and emergency department use despite comparable levels of outpatient care [4].

Taken together, these findings indicate that inequities in ALOS persist despite the equity principles of the Canada Health Act [1]. From a public health perspective, disparities arise not only from social and geographic disadvantage, but also from system-level constraints that limit responsiveness during periods of acute demand. Addressing these gaps requires strengthening primary and preventive care, improving coordination during urgent admissions, and alleviating capacity pressures in high-volume hospitals. Such system-wide interventions are essential to reducing avoidable variation in hospital length of stay, improving public health outcomes, and advancing equity in Canada’s health system.

By explicitly examining interaction effects, this study extends existing evidence by showing how admission urgency modifies socioeconomic and geographic gradients in ALOS, revealing patterns that are not apparent from main effects alone. While many associations were statistically significant due to the large sample size, the magnitude of these effects represents meaningful differences in hospital resource use, as even modest proportional increases in ALOS translate into substantial cumulative system burden at the population level. These findings highlight the importance of considering both patient characteristics and admission context in understanding variation in hospital length of stay, particularly in a publicly funded system where demand pressures may differentially affect subgroups.

These results have clear policy relevance. Interventions aimed at improving timely access to primary and specialist care, particularly for lower-income and rural populations, may reduce the need for urgent admissions that are associated with longer stays. The findings also point to the importance of system responsiveness during high-acuity episodes, where existing socioeconomic and geographic advantages appear to diminish. Methodologically, the analysis used a negative binomial generalized estimating equations model with clustering at the admission level, ensuring robust inference. Finally, ALOS in this study reflects time spent in acute care beds only, with days spent awaiting transfer to other care settings explicitly excluded. As such, the measure accurately captures acute hospital resource use rather than total length of stay and does not represent an underestimation of acute care duration.

## 6. Limitations

This analysis has several limitations. First, the use of administrative data precludes direct measurement of clinical severity, education, and marital status, all of which may influence length of stay. Second, socioeconomic status was measured using neighborhood-level income quintiles, which may mask important variation at the individual level. Third, rural–urban status reflects patients’ place of residence rather than the location of the hospital providing care, and high-acuity rural patients may be treated in urban hospitals, complicating interpretation of geographic effects. Finally, associations attributed to urban residence may partially reflect hospital-level characteristics, such as teaching hospitals, which account for approximately 6% of all hospitals, rather than geographic context alone.

## 7. Conclusions

This study demonstrates that admission urgency, socioeconomic status, sex, and geography jointly shape acute length of stay in Canada beyond clinical need alone. While higher income and urban residence were generally associated with shorter stays, these advantages were diminished under urgent admissions, when ALOS increased substantially across all social groups. This pattern highlights how inequities in hospital use reflect not only differences in patient characteristics but also broader social and system-level constraints.

By demonstrating how admission urgency modifies social and geographic gradients in ALOS, this study adds to the understanding of equity within Canada’s universal healthcare system. Despite the principles of the Canada Health Act, disparities persist, particularly during periods of acute demand. From a public health perspective, reducing avoidable variation in hospital length of stay requires policies that extend beyond hospital-based care to strengthen timely access to primary and specialist services, improve care coordination before conditions become urgent, and address persistent geographic and socioeconomic barriers. Upstream and system-level interventions of this kind are essential for improving efficiency, reducing preventable hospital use, and advancing equity in Canada’s health system.

## Figures and Tables

**Table 1:** Descriptive statistics (mean and standard deviation) of continuous variables used in regression model.

	Mean	Standard Deviation
Dependent variable: ALOS (in days)	7.09	12.06
Age	60.70	23.34
Number of comorbid conditions	3.21	3.25
Number of observations	1,851,045	

**Table 2:** Frequency distribution of categorical variables used in regression model.

	Frequency	Percentage
**Sex**		
Female	929,332	50.21
Male	921,713	49.79
**Admission Type**		
Elective	432,562	23.37
Urgent	1,418,483	76.63
**Residence (area)**		
Rural	359,131	19.40
Urban	1,491,914	80.60
**Neighbourhood Income Quintile**		
Quintile 1 (lowest)	499,756	27.00
Quintile 2	405,884	21.93
Quintile 3	344,363	18.60
Quintile 4	311,267	16.82
Quintile 5	289,775	15.65
**Primary diagnosis**		
Infectious and parasitic diseases	65,886	3.56
Neoplasms	131,007	7.08
Diseases of the blood	22,986	1.24
Endocrine and related diseases	67,261	3.63
Mental and behavioral disorders	119,128	6.44
Diseases of the nervous system	42,969	2.32
Diseases of the circulatory system	263,345	14.23
Diseases of the respiratory system	198,713	10.74
Diseases of the digestive system	232,226	12.55
Diseases of the skin and subcutaneous tissue	26,064	1.41
Diseases of the musculoskeletal system and connective tissue	148,063	8.00
Diseases of the genitourinary system	120,187	6.49
Symptoms, signs, and abnormal clinical and laboratory findings	117,836	6.37
Injury and poisoning	191,897	10.37
Factor influencing health status	103,477	5.59
**Province**		
Newfoundland	38,332	2.07
Prince Edward Island	10,495	0.57
Nova Scotia	71,805	3.88
New Brunswick	58,130	3.14
Ontario	895,991	48.4
Manitoba	78,710	4.25
Saskatchewan	91,828	4.96
Alberta	268,424	14.5
British Columbia	337,330	18.22

Note: Totals may not sum exactly due to rounding.

**Table 3:** Partial results of the Negative Binomial Regression with interactive terms, 2024–2025.

	Estimate	Robust Std. Error	Wald 95% Confidence Limits		IRRs	Combined IRRs ***
Intercept	1.238 **	0.0101	1.2184	1.2581		
Age	0.004 **	0.000	0.0042	0.0044	1.004	
Male (Ref: Female)	0.095 **	0.0051	0.0847	0.1048	1.099	
Urban (Ref: Rural)	−0.063 **	0.0067	−0.0762	−0.0499	0.939	
Income Quintile 1 (lowest)	Ref.				1.000	
Income Quintile 2	−0.050 **	0.008	−0.0652	−0.034	0.952	
Income Quintile 3	−0.068 **	0.0085	−0.0843	−0.0511	0.935	
Income Quintile 4	−0.097 **	0.0074	−0.1116	−0.0827	0.907	
Income Quintile 5	−0.131 **	0.0072	−0.1451	−0.117	0.877	
Urgent (Ref: Elective)	0.278 **	0.009	0.2602	0.2953	1.320	
Income Quintile 2 × Urgent	0.0315 **	0.0084	0.0151	0.048	1.032	1.297
Income Quintile 3 × Urgent	0.0407 **	0.0089	0.0233	0.0582	1.042	1.285
Income Quintile 4 × Urgent	0.0656 **	0.0079	0.0501	0.0812	1.068	1.279
Income Quintile 5 × Urgent	0.0949 **	0.0078	0.0797	0.1101	1.100	1.273
Urban × Urgent	0.051 **	0.0071	0.0371	0.0649	1.052	1.304
Sex × Urgent	−0.126 **	0.0054	−0.1366	−0.1153	0.882	1.279
Number of comorbid conditions	0.166 **	0.0003	0.1651	0.1662	1.180	
Dispersion	0.594	0.001	0.592	0.595		
Deviance: Value/Degree of freedom	0.977					
Number of observations	1,851,045					

Note: ** *p* < 0.01. *** Example: The combined IRR for *Income Quintile 2 × Urgent* is calculated as: exp(*coef. Income Quintile 2 + coef. Urgent Admission + coef. Interaction*) = *e*xp(−0.050 + 0.278 + 0.032) = 1.297. This corresponds to a (1.297 − 1) × 100 = 29.7% increase in expected ALOS. Detailed results for primary diagnosis categories and provincial indicators are provided in Table A3.

**Table 4:** Adjusted ALOS by key patient subgroups.

Subgroup	Category	Adjusted ALOS (Days)	Relative Difference vs. Reference (%)	95% CI
Sex	Female	6.8	Ref.	6.8–6.9
	Male	7.5	+10%	7.4–7.6
Residence	Rural	7.3	Ref.	7.2–7.4
	Urban	6.9	−6%	6.8–7.0
Income Quintile	Q1 (lowest)	7.6	Ref.	7.5–7.7
	Q2	7.3	−4%	7.2–7.4
	Q3	7.1	−6%	7.0–7.2
	Q4	6.9	−9%	6.8–7.0
	Q5 (highest)	6.7	−12%	6.6–6.8
Admission Category	Elective	6.8	Ref.	6.7–6.9
	Urgent	9.0	+32%	8.9–9.1

Notes: Adjusted ALOS values are predicted from the negative binomial model controlling for age, comorbid conditions, province, and primary diagnosis. Relative differences are calculated as the percent change compared with the reference category within each subgroup.

**Table 5:** Adjusted ALOS by Key Interaction Subgroups.

Interaction	Subgroup	Adjusted ALOS (Days)	Relative Difference vs. Reference (%)	95% CI
Sex × Admission Category	Female-Elective	6.8	Ref.	6.7–6.9
	Female-Urgent	9.0	+32%	8.9–9.1
	Male-Elective	7.5	+10%	7.4–7.6
	Male-Urgent	8.9	+31%	8.8–9.0
Residence (area) × Admission Category	Rural-Elective	7.3	Ref.	7.2–7.4
	Rural-Urgent	9.1	+25%	9.0–9.2
	Urban-Elective	6.9	−6%	6.8–7.0
	Urban-Urgent	9.6	+32%	9.5–9.7
Neighbourhood Income Quintile × Admission Category	Quintile1-Elective	7.6	Ref.	7.5–7.7
	Quintile1-Urgent	9.2	+21%	9.1–9.3
	Quintile2-Elective	7.3	−4%	7.2–7.4
	Quintile2-Urgent	9.0	+18%	8.9–9.1
	Quintile3-Elective	7.1	−6%	7.0–7.2
	Quintile3-Urgent	8.9	+18%	8.8–9.0
	Quintile4-Elective	6.9	−9%	6.8–7.0
	Quintile4-Urgent	8.9	+18%	8.8–9.0
	Quintile5-Elective	6.7	−12%	6.6–6.8
	Quintile5-Urgent	9.0	+19%	8.9–9.1

Notes: Adjusted ALOS values are predicted from the negative binomial model controlling for age, comorbid conditions, province, and primary diagnosis. Relative differences are percent changes relative to the reference category within each interaction. Urgent admissions substantially increase ALOS across all subgroups, but the magnitude varies by sex, area, and income.

## Data Availability

Health Canada obtained the data from the Canadian Institute for Health Information (CIHI). Health Canada is authorized to make the data available to its employees. However, they are not allowed to share the data. One can request CIHI for the data.

## Appendix A

**Table A1:** Descriptive statistics (mean and standard deviation) of continuous variables used in regression models by admission type.

	Elective Admission		Urgent Admission	
	Mean	Standard Deviation	Mean	Standard Deviation
ALOS (in days)	4.59	11.02	7.85	12.26
Age	59.85	20.42	60.96	24.15
Number of comorbid conditions	1.99	2.49	3.59	3.36
Number of observations	432,562		1,418,483	

**Table A2:** Frequency Distribution of categorical variables used in regression models by admission type.

	Elective Admission N = 432,562		Urgent Admission N = 1,418,483	
**Sex**	**Frequency**	**Percentage**	**Frequency**	**Percentage**
Female	229,961	53.16	699,371	49.30
Male	202,601	46.84	719,112	50.70
**Residence (Area)**				
Rural	88,903	20.55	270,228	19.05
Urban	343,659	79.45	1,148,255	80.95
**Income Quintile**				
Quintile 1 (lowest)	96,337	22.27	403,419	28.44
Quintile 2	91,560	21.17	314,324	22.16
Quintile 3	83,860	19.39	260,503	18.36
Quintile 4	80,476	18.6	230,791	16.27
Quintile 5	80,329	18.57	209,446	14.77
**Primary Diagnosis**				
Infectious and parasitic diseases	1944	0.45	63,942	4.51
Neoplasms	82,612	19.1	48,395	3.41
Diseases of the blood	1420	0.33	21,566	1.52
Endocrine and related diseases	14,060	3.25	53,201	3.75
Mental and behavioral disorders	6841	1.58	112,287	7.92
Diseases of the nervous system	8932	2.06	34,037	2.40
Diseases of the circulatory system	41,023	9.48	222,322	15.67
Diseases of the respiratory system	10,331	2.39	188,382	13.28
Diseases of the digestive system	30,547	7.06	201,679	14.22
Diseases of the skin and subcutaneous tissue	1441	0.33	24,623	1.74
Diseases of the musculoskeletal system and connective tissue	106,675	24.66	41,388	2.92
Diseases of the genitourinary system	38,479	8.9	81,708	5.76
Symptoms, signs, and abnormal clinical and laboratory findings	6657	1.54	111,179	7.84
Injury and poisoning	22,332	5.16	169,565	11.95
Factor influencing health status	59,268	13.7	44,209	3.12
**Province**				
Newfoundland	9012	2.08	29,320	2.07
Prince Edward Island	2458	0.57	8037	0.57
Nova Scotia	16,770	3.88	55,035	3.88
New Brunswick	13,131	3.04	44,999	3.17
Ontario	205,448	47.5	690,543	48.68
Manitoba	17,038	3.94	61,672	4.35
Saskatchewan	22,702	5.25	69,126	4.87
Alberta	62,946	14.55	205,478	14.49
British Columbia	83,057	19.2	254,273	17.93

Note: Totals may not sum exactly due to rounding.

**Table A3:** Results of the Negative Binomial Regression with interactive terms, 2024–2025. (continuation of Table 3.

	Estimate	Robust Std. Error	Wald 95% Confidence Limits		IRRs
Infectious and parasitic diseases	−0.680 **	0.0003	−0.6916	−0.6676	0.507
Neoplasms	−0.460 **	0.0003	−0.4718	−0.4476	0.631
Diseases of the blood	−0.718 **	0.0003	−0.7349	−0.7000	0.488
Endocrine and related diseases	−0.698 **	0.0003	−0.7127	−0.6841	0.497
Mental and behavioral disorders	Ref.				1.000
Diseases of the nervous system	−0.361 **	0.0003	−0.3785	−0.3444	0.697
Diseases of the circulatory system	−0.675 **	0.0003	−0.6853	−0.6653	0.509
Diseases of the respiratory system	−0.697 **	0.0003	−0.7066	−0.6867	0.498
Diseases of the digestive system	−0.716 **	0.0003	−0.7263	−0.706	0.489
Diseases of the skin and subcutaneous tissue	−0.442 **	0.0003	−0.4582	−0.4256	0.643
Diseases of the musculoskeletal system and connective tissue	−0.814 **	0.0003	−0.8266	−0.8004	0.443
Diseases of the genitourinary system	−0.961 **	0.0003	−0.9717	−0.9493	0.383
Symptoms, signs, and abnormal clinical and laboratory findings	−0.847 **	0.0003	−0.8583	−0.8353	0.429
Injury and poisoning	−0.644 **	0.0003	−0.6546	−0.6337	0.525
Factor influencing health status	−0.488 **	0.0003	−0.5033	−0.4725	0.614
Newfoundland	0.345 **	0.0067	0.3323	0.3585	1.413
Prince Edward Island	0.414 **	0.0151	0.3843	0.4434	1.513
Nova Scotia	0.298 **	0.0051	0.2878	0.3077	1.347
New Brunswick	0.367 **	0.0053	0.3571	0.3777	1.444
Ontario	Ref.				1.000
Manitoba	0.549 **	0.0049	0.5393	0.5584	1.731
Saskatchewan	0.427 **	0.0045	0.4183	0.436	1.533
Alberta	0.308 **	0.0028	0.3024	0.3133	1.361
British Columbia	0.300 **	0.0025	0.2954	0.3053	1.350
Dispersion	0.594	0.001	0.592	0.595	
Deviance: Value/Degree of freedom	0.977				
Number of observations	1,851,045				

Note: ** *p* < 0.01.
